# Handgrip Strength Thresholds to Detect Cardiometabolic Risk in Youth: Cross‐Sectional Study and Meta‐Analysis

**DOI:** 10.1002/jcsm.70091

**Published:** 2025-10-20

**Authors:** Antonio García‐Hermoso, Rodrigo Yáñez‐Sepúlveda, Ignacio Hormazábal‐Aguayo, Jacinto Muñoz‐Pardeza, Vicente Martínez‐Vizcaíno, Juan Hurtado‐Almonacid, Yasmin Ezzatvar

**Affiliations:** ^1^ Navarrabiomed, Hospital Universitario de Navarra, Universidad Pública de Navarra (UPNA), IdiSNA Pamplona Spain; ^2^ Faculty of Education and Social Sciences Universidad Andres Bello Viña del Mar Chile; ^3^ School of Medicine Universidad Espíritu Santo (UEES) Samborondón Ecuador; ^4^ Vicerrectoría de Investigación y Postgrado School of Medicine, Universidad Espíritu Santo (UEES) La Serena Chile; ^5^ Health and Social Research Center Universidad de Castilla‐La Mancha Cuenca Spain; ^6^ Facultad de Ciencias de la Salud Universidad Autónoma de Chile Talca Chile; ^7^ eFidac grupo de investigación. Escuela de Educación Física Pontificia Universidad Católica de Valparaíso Valparaíso Chile; ^8^ Lifestyle factors with impact on Ageing and overall Health (LAH) Research Group. Department of Nursing University of València Valencia Spain; ^9^ Vicerrectoría de Investigación y Postgrado Universidad de Los Lagos Osorno Chile

**Keywords:** cut‐off, grip strength, metabolic syndrome, receiver operating characteristic curves

## Abstract

**Background:**

Muscular fitness, particularly handgrip strength, is increasingly recognized as a robust marker of cardiometabolic risk (CMR) in children and adolescents. However, evidence‐based diagnostic thresholds for identifying at‐risk individuals remain scarce, particularly in children. This study aimed to (1) establish sex‐specific diagnostic thresholds for handgrip strength normalized to body weight to identify elevated CMR in children aged 8–11 years, and (2) synthesize existing evidence through a systematic review and meta‐analysis across pediatric age groups, integrating the new data with existing evidence.

**Methods:**

We analyzed cross‐sectional data from 1124 Spanish children (49.7% girls) aged 8–11 years participating in the MOVI‐2 study. Normalized handgrip strength was associated with a CMR index composed of waist circumference, triglyceride‐to‐HDL ratio, mean arterial pressure and fasting insulin. Diagnostic accuracy was assessed using receiver operating characteristic curves and optimized with the Youden Index. Results from the MOVI‐2 study and other diagnostic accuracy studies were combined in a meta‐analysis for identifying the optimal threshold for normalized handgrip strength to identify elevated CMR in youth.

**Results:**

In the MOVI‐2 study, thresholds were 0.38 for boys and 0.34 for girls, with area under the curve (AUC) of 0.77 (95% CI: 0.73–0.81) and 0.75 (95% CI: 0.70–0.79), respectively. The systematic review and meta‐analysis followed PRISMA‐DTA guidelines and included nine additional studies (*n* = 10 588). Meta‐analytic thresholds for normalized handgrip strength were 0.30 for girls and 0.39 for boys in childhood (6–12 years), and 0.36 for girls and 0.42 for boys in adolescence (13–18 years), with the highest diagnostic accuracy observed in adolescent girls (AUC = 0.80, 95% CI: 0.77–0.83; Youden Index = 0.60). Children showed greater heterogeneity, particularly in specificity.

**Conclusions:**

Despite certain limitations, our findings provide clinically relevant, sex‐ and age‐specific thresholds for normalized handgrip strength to identify elevated CMR in youth. These thresholds may serve as a valuable starting point for CMR screening in both boys and girls.

## 1 Introduction

Cardiometabolic risk (CMR) factors, such as abdominal obesity, dyslipidemia, hypertension and insulin resistance, are increasingly prevalent among children and adolescents, raising concerns for future cardiovascular and metabolic diseases [1]. Timely identification of children and adolescents with elevated CMR is critical to enable early prevention and limit the progression of chronic disease into adulthood [2].

Within this context, muscular fitness has gained recognition as a critical indicator of health in pediatric populations [3]. Growing evidence suggests that higher levels of muscular fitness during childhood are associated with a lower risk of developing metabolic syndrome in adulthood, as well as with reduced continuous CMR scores later in life [4]. Although muscular fitness is widely recognized as a key health marker in children and adolescents, the absence of standardized, evidence‐based cut‐points for strength limits its utility in surveillance and intervention. These thresholds are crucial for identifying at‐risk individuals and guiding public health strategies, yet few studies have assessed their predictive validity using clinical metrics like sensitivity or specificity [5, 6].

Handgrip strength, in particular, is a simple, non‐invasive and cost‐effective measure of overall muscular fitness that has been inversely associated with CMR factors [5]. Recent studies suggest that handgrip strength normalized by body mass may serve as a useful indicator for identifying youth at higher CMR [6]. However, further research is needed to establish age‐ and sex‐specific cut‐off points to support its clinical application. Specifically, there is a lack of data in children aged 6–12 years [7], which limits the generalizability of existing thresholds and delays early identification efforts in this critical developmental period [5]. In particular, the ages between 8 and 11 years represent a key window for cardiometabolic screening, as this stage often precedes the onset of puberty, when physiological and hormonal changes begin to accelerate [2]. To date, only one meta‐analysis has attempted to determine a handgrip strength cut‐off point [6]; however, methodological limitations in that study, such as the combined analysis of data from children and adolescents and the inclusion of duplicated data from the same databases, highlight the need for complementary approaches. Therefore, the present study aimed to: (a) establish and evaluate sex‐specific diagnostic thresholds of normalized handgrip strength for identifying CMR in children aged 8–11 years using a large sample; and (b) synthesize existing evidence through a systematic review and meta‐analysis to assess the overall accuracy and consistency of such thresholds across different ages and between sexes.

## 2 Method

### 2.1 Cross‐Sectional Analysis

#### 2.1.1 Study Design and Participants

This study involved a cross‐sectional analysis of baseline data collected between September and November 2010 as part of a cluster‐randomized trial evaluating the effectiveness of a school‐based physical activity intervention (MOVI‐2) designed to prevent excessive weight gain in children [8]. The MOVI‐2 project enrolled 1158 children aged 8–11 years from 20 public primary schools in the province of Cuenca, Spain. Ethical approval was granted by the Clinical Research Ethics Committee of Virgen de la Luz Hospital in Cuenca. Following approval from each school's Director and School Board (Consejo Escolar), invitation letters were sent to parents of 4th and 5th grade students, inviting them to an informational meeting. During this meeting, the study objectives were explained, and written consent for the children's participation was requested. Subsequently, informative sessions were held class by class to obtain verbal assent from the students. The final sample for the present analysis included 1124 children (49.7% girls).

#### 2.1.2 Anthropometric and Cardiometabolic Assessments

All assessments were conducted at the schools by qualified nurses specifically trained for the procedures.

### 2.2 Anthropometric and Functional Measures

Weight and height were each measured twice, with a 5‐min interval between measurements. Body weight was recorded to the nearest 100 g using a calibrated digital scale (SECA Model 861; Vogel & Halke, Hamburg, Germany), with children wearing light clothing and no shoes. Height was assessed to the nearest millimeters using a wall‐mounted stadiometer, with participants standing upright against the wall, barefoot and with the spine aligned to the stadiometer. The head was positioned with the chin parallel to the floor. The average of the two measurements was used to calculate body mass index (BMI), defined as weight in kilograms divided by height in meters squared (kg/m^2^). Waist circumference (WC) was obtained as the mean of two measurements taken at the natural waist, midway between the last rib and the iliac crest, using a flexible measuring tape.

### 2.3 Blood Pressure

Systolic blood pressure (SBP) and diastolic blood pressure (DBP) were measured twice, with a 5‐min interval between readings. Prior to the first measurement, children rested for a minimum of 5 min. Assessments were conducted in a quiet, relaxed setting, with participants seated and the right arm positioned semi‐flexed at heart level. Blood pressure was recorded using an automated device (OMRON M5‐I; Omron Healthcare Europe BV, Hoofddorp, Netherlands). Mean arterial pressure was derived using the formula: DBP + 0.333 × (SBP − DBP).

### 2.4 Biochemical Assessments

Blood samples were collected under standardized conditions between 8:15 and 9:00 AM, following a minimum 12‐h overnight fast. Fasting status was confirmed by both the child and their parents prior to venipuncture, which was performed via the cubital vein. When transportation to the laboratory was expected to exceed 75 min, samples were centrifuged on‐site and transported under refrigerated conditions. Three aliquots were prepared from each sample: one was used for the biochemical analyses included in this study, while the remaining two were stored for future research purposes, as disclosed to the parents [8]. Samples were processed and analyzed for glucose, insulin, total cholesterol, high‐density lipoprotein cholesterol (HDL‐C), low‐density lipoprotein cholesterol (LDL‐C) and triglycerides using standardized clinical laboratory methods.

### 2.5 CMR Score

A CMR index was computed by summing the age‐ and sex‐standardized z‐scores of WC, the triglyceride‐to‐HDL cholesterol ratio, mean arterial blood pressure and fasting insulin levels. The construct validity of this index has been previously supported through confirmatory factor analysis [9]. Children with a composite score ≥ 1 standard deviation were classified as having elevated CMR.

#### 2.5.1 Handgrip Strength Assessment

Maximum handgrip strength was measured using a TKK 5401 Grip‐D dynamometer (Takei, Tokyo, Japan), which has a measurement range of 5–100 kg and a precision of 0.1 kg. The grip span of the device was adjusted to fit each child's hand size. Participants were instructed to fully extend their elbow and squeeze the dynamometer with their right hand for at least 2 s, followed by the same procedure with the left hand. Each hand was tested twice, and the highest value from each hand was recorded in kilograms. For analysis, the average of the maximum values from both hands was used. Handgrip was normalized by dividing absolute handgrip strength (in kg) by body weight (in kg).

#### 2.5.2 Statistical Analysis

To identify optimal diagnostic thresholds, we used receiver operating characteristic (ROC) curve analyses to estimate the cut‐off points of normalized handgrip strength associated with elevated CMR. For each diagnostic variable, sensitivity and specificity were computed across a range of thresholds, and the area under the ROC curve (AUC) was used to evaluate the discriminative ability of handgrip strength. The optimal threshold was defined as the point maximizing the Youden Index (sensitivity + specificity − 1). In addition, we calculated the diagnostic odds ratio (dOR), positive likelihood ratio (PLR) and negative likelihood ratio (NLR), which provide insight into the strength and direction of the association between the test result and CMR. Furthermore, we assessed the positive predictive value (PPV) and negative predictive value (NPV) to estimate the test's practical utility in identifying true cases and non‐cases within the sample.

### 2.6 Systematic Review and Meta‐Analysis

The systematic review and meta‐analysis was carried out in accordance with the Preferred Reporting Items for Systematic Reviews and Meta‐Analyses of Diagnostic Test Accuracy Studies (PRISMA‐DTA) guidelines [10]. Also, the study was registered in the International Prospective Register of Systematic Reviews (PROSPERO: CRD420251064696).

#### 2.6.1 Eligibility Criteria

Studies were required to meet the following criteria: (1) Participants: apparently healthy preschoolers, children and adolescents aged 3–18 years (or with a mean age below 18), or general population studies that provided data on a relevant pediatric subgroup; (2) Exposure: studies reporting handgrip strength normalized by body mass as the main exposure variable; (3) Outcome: composite indicators of CMR (e.g., metabolic syndrome or aggregated risk scores); (4) Study design: observational studies including cross‐sectional and cohort (prospective or retrospective) designs. Only peer‐reviewed articles published in English were included.

#### 2.6.2 Information Source and Search

A comprehensive literature search was performed across four major databases, MEDLINE (via PubMed), EMBASE, Web of Science and SPORTDiscus, from their inception until May 2025. Details of the search strategy are provided in Table S1. Additionally, reference lists of relevant primary studies and prior systematic reviews were manually screened to identify any potentially eligible studies not captured in the electronic search.

#### 2.6.3 Study Selection

After identifying the studies in the four databases, Zotero reference manager (Version 5.0, George Mason University, Fairfax, VA, USA) was used to remove duplicates.

#### 2.6.4 Data Collection Process

We extracted the following data from each included study: (1) study characteristics, including first author's name, year of publication, country or region of the study, sample size and study design; (2) participant information, such as age and sex; (3) prevalence of CMR or metabolic syndrome; (4) diagnostic accuracy indicators, including sensitivity and specificity; and (5) the optimal normalized handgrip strength thresholds identified in relation to CMR.

#### 2.6.5 Definitions for Data Extraction

A standardized data extraction table was organized by age group (i.e., children, adolescents and mixed samples including both) and by sex. Reported diagnostic accuracy parameters were used to derive the values of true positives (TP), false positives (FP), true negatives (TN) and false negatives (FN) for each study.

#### 2.6.6 Risk of Bias and Applicability

The methodological quality of the included studies was independently assessed by two reviewers using the Quality Assessment of Diagnostic Accuracy Studies‐2 (QUADAS‐2) tool [11]. This instrument assesses the risk of bias and applicability concerns in diagnostic accuracy studies across four domains: patient selection, index test, reference standard, and flow and timing.

#### 2.6.7 Diagnostic Accuracy Measures

To evaluate the performance of each diagnostic threshold, sensitivity and specificity were calculated and logit‐transformed to stabilize variance and meet normality assumptions required for regression analysis. The Youden Index, defined as (sensitivity + specificity − 1), was used as the criterion for identifying the optimal threshold, representing the point that maximizes combined diagnostic accuracy [12].

From two studies [13, 14], TP, FP, TN and FN were estimated using the sample size, the proportion of participants classified as at‐risk by normalized handgrip strength and the proportion identified with CMR based on z‐score thresholds. The number of correctly classified cases (TP + TN) was derived from the reported correct classification rate. From these values, a system of equations was applied to estimate the distribution of TP, FP, TN and FN in each subgroup. This method aligns with established approaches for reconstructing diagnostic contingency tables from summary data when individual‐level data is not available [15].

#### 2.6.8 Synthesis of Results

For each of the four demographic subgroups (girls and boys, children and adolescents), the relationship between the diagnostic threshold and test performance was evaluated. Separate analyses were conducted to examine how variations in threshold levels influence sensitivity and specificity across studies. These estimates were pooled and summarized to support subgroup comparisons and facilitate interpretation of diagnostic utility within each group [16].

#### 2.6.9 Meta‐Analysis

Random‐effects meta‐analysis models were fitted independently for each demographic subgroup using the *metafor* package in R (Version 4.5.0) [17]. Two models were estimated per group, one for logit‐transformed sensitivity and another for logit‐transformed specificity, considering the diagnostic threshold as a continuous moderator. This modelling approach accounted for both within‐study sampling error and between‐study heterogeneity. Although HSROC modelling was initially considered, it was ultimately not included in the final analysis. Given that the diagnostic threshold was treated as a continuous moderator and the primary objective was to determine optimal cut‐points through numerical optimization (i.e., maximizing the Youden Index), the use of HSROC was deemed redundant. The chosen approach allowed for more direct and clinically meaningful estimation of subgroup‐specific thresholds while still accounting for between‐study heterogeneity.

#### 2.6.10 Additional Analyses

Numerical optimization procedures were implemented to identify the threshold that maximized the Youden Index for each subgroup. This approach enabled the determination of the diagnostic threshold associated with the highest overall accuracy. All analyses were conducted using R, with statistical significance set at *p* < 0.05.

## 3 Results

### 3.1 Cross‐Sectional (MOVI‐2)

Table 1 shows that boys had significantly higher HDL‐C (61.95 ± 13.98 mg/dL vs. 59.01 ± 12.91 mg/dL, *p* < 0.001), SBP (102.65 ± 9.38 mmHg vs. 99.61 ± 9.04 mmHg, *p* < 0.001) and normalized handgrip strength (0.421 ± 0.091 vs. 0.381 ± 0.079, *p* < 0.001), while girls had higher insulin (8.59 ± 4.98 μU/mL vs. 7.35 ± 4.95 μU/mL, *p* < 0.001) and triglycerides (69.58 ± 35.06 mg/dL vs. 63.01 ± 33.52 mg/dL, *p* = 0.001).

**TABLE 1:** Characteristics of the MOVI‐2 sample.

	Boys		Girls		*p*‐value
	Mean	SD	Mean	SD	
Age	9.51	0.73	9.48	0.69	0.479
Body mass index, kg/m^2^	19.16	3.82	18.85	3.55	0.142
Waist circumference, cm	68.24	9.73	67.03	8.98	0.03
HDL cholesterol, mg/dL	61.95	13.98	59.01	12.91	< 0.001
Triglycerides, mg/dL	63.01	33.52	69.58	35.06	0.001
Systolic BP, mmHg	102.65	9.38	99.61	9.04	< 0.001
Diastolic BP, mmHg	62.43	7.3	62.35	7.13	0.848
Glucose, mg/dL	84.66	7.66	82.46	6.13	< 0.001
Insulin, μU/mL	7.35	4.95	8.59	4.98	< 0.001
Triglycerides‐to‐HDL ratio	1.14	0.88	1.31	0.96	0.002
Mean arterial pressure, mmHg	75.84	7.32	74.77	7.10	0.012
CMR index	−0.007	1.7	−0.004	1.71	0.977
CMR ≥ 1 SD, *n* (%)	157 (27.8)		148 (26.5)		0.621
Handgrip strength, kg	15.38	3.46	13.79	3.09	< 0.001
Normalized handgrip strength	0.42	0.09	0.38	0.08	< 0.001

Abbreviations: BP, blood pressure; CMR, cardiometabolic risk; HDL, high density lipoprotein; SD, standard deviation.

**FIGURE 1:**
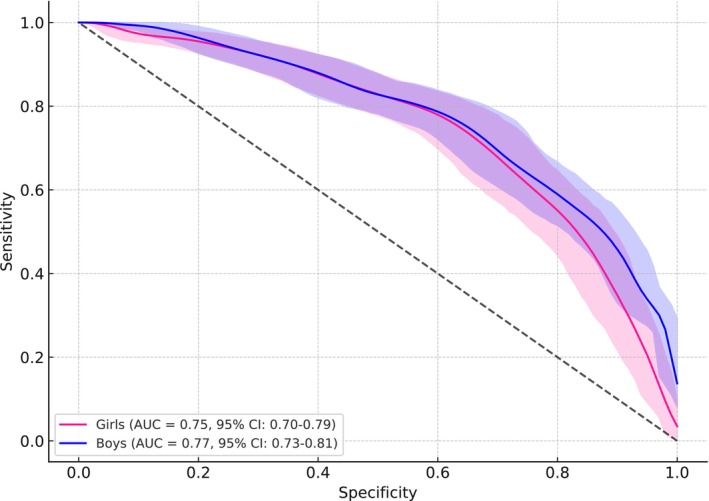
Receiver operating characteristic curves illustrating the diagnostic performance of normalized handgrip strength in predicting cardiometabolic risk among boys and girls.

Figure 1 presents the ROC curves illustrating the discriminative ability of normalized handgrip strength for predicting elevated CMR in boys and girls. Among boys, the AUC was 0.77 (95% CI: 0.73–0.81), with an optimal cut‐off point of 0.38, yielding a sensitivity of 67% and specificity of 75%. These values correspond to a dOR of 6.09, a PLR of 2.68 and an NLR of 0.44. The PPV was 50.8%, and the NPV was 85.5%. In girls, the AUC was 0.75 (95% CI: 0.70–0.79), with an optimal threshold of 0.34, associated with 65% sensitivity and 74% specificity. This yielded a dOR of 5.28, a PLR of 2.50 and an NLR of 0.47. The PPV and NPV were 47.4% and 85.4%, respectively.

### 3.2 Systematic Review and Meta‐Analysis

#### 3.2.1 Study Selection

The systematic search identified 1283 records, of which 15 full‐text articles were assessed for eligibility. After excluding six studies (Supporting Information S1: eMethod 1), nine were included in the systematic review and meta‐analysis [13, 14, 18, 19, 20, 21, 22, 23], along with our own, the MOVI‐2 study (Figure S1).

#### 3.2.2 Study Characteristics

The total sample across the included studies comprised 10 588 participants, of whom 5335 were girls, representing approximately 50.4% of the total sample. Sample sizes ranged from 452 [21] to 2303 [20] participants, with age ranges between 6 and 18 years. Most studies assessed normalized handgrip strength (normalized by body weight) and used components of the metabolic syndrome to define CMR (Table 2).

**TABLE 2:** Summary of the included studies' characteristics.

Study/country	Sample size	Age range	Design	Handgrip assessment	CMR definition	Participants at risk	Cut‐off
Castro‐Piñero et al. [19] Spain	511 (270 boys, 241 girls)	6–17	Cross‐sectional and longitudinal (Up and Down)	Handgrip strength was assessed using a TKK 5101 Grip D dynamometer (Takei, Tokyo, Japan) with adjustable grip‐span according to hand size, following child‐ and adolescent‐specific equations. The test was performed twice per hand; the maximum value for each hand was recorded, and the average of both hands was used.	The variables included in the CMR score were sum of two skinfolds; SBP, HOMA‐IR, TG and TC/HDL‐C ratio	Children Boys: 15.7% Girls: 15.5% Adolescents Boys: 15.5% Girls: 15.4%	Children Boys: 0.37 Girls: 0.31 Adolescents Boys: 0.47 Girls: 0.42
Castro‐Piñero et al. [18] Europe (9 countries)	969 (449 boys, 520 girls)	12–17	Cross‐sectional (HELENA)	Handgrip strength was measured using a TKK 5101 Grip D dynamometer (Takei, Tokyo, Japan) with grip‐span adjusted to hand size. Adolescents performed two trials per hand (elbow extended, ≥ 2 s squeeze), with 1‐min rest between trials. The maximum value from each hand was recorded, and their average was used for analysis.	All CMR factors were standardized as age‐ and sex‐specific z‐scores based. A continuous CMR index was calculated as the average z‐score of WC, mean arterial pressure, TG/HDL ratio and fasting insulin.	Adolescents Boys: 6.8% Girls: 7.5%	Adolescents Boys: 0.52 Girls: 0.41
DeHondt et al. [13] USA	1033 (535 boys, 498 girls)	12–17	Cross‐sectional (NHANES)	Handgrip strength was measured with a Takei TKK 5401 dynamometer, adjusted to hand size. The test followed the Eurofit protocol, with three trials per hand. The highest value of the dominant hand was used.	A continuous CMR score was calculated as the sum of z‐scores for WC, triglycerides, HDL‐C (multiplied by −1), glucose and mean arterial pressure.	Adolescents Boys: 17.0% Girls: 11.3%	Adolescents Boys: 0.39 Girls: 0.34
Ko and Kim [22] South Korea	920 (496 boys, 424 girls)	10–12	Cross‐sectional (KNHANES 2014–2018)	Handgrip strength was measured with a TKK 5401 dynamometer following ACSM protocol. Three trials per hand were performed; the best value was used.	Metabolic syndrome diagnosed using Cook et al. criteria: ≥ 3 of the following—waist circumference ≥ 90th percentile, BP ≥ 90th percentile, TG ≥ 110 mg/dL, HDL‐C ≤ 40 mg/dL, glucose ≥ 110 mg/dL, or use of medication for BP, lipids or diabetes.	Children Boys: 3.2% Girls: 1.6% Adolescents 13–15 years Boys: 7.10% Girls: 3.9% Adolescents 16–18 years Boys: 6.4% Girls: 4.9%	Children Boys: 0.35 Girls: 0.37 Adolescents 13–15 years Boys: 0.47 Girls: 0.38 Adolescents 16–18 years Boys: 0.49 Girls: 0.38
Lee et al. [20] South Korea	2303 (1226 boys, 1077 girls)	13–18	Cross‐sectional (KNHANES 2014–2017)	Handgrip was measured with a Takei TKK 5401 dynamometer in standing position. Three trials per hand were performed with 60 s rest; the highest value from each hand was recorded.	Metabolic syndrome defined using IDF criteria: for ages 10–15, ≥ 3 of the following—WC ≥ 90th percentile, TG ≥ 150 mg/dL, HDL < 40 mg/dL, SBP ≥ 130 mmHg or DBP ≥ 85 mmHg, glucose ≥ 100 mg/dL. For ≥ 16 years, WC ≥ 90 cm (boys) or ≥ 80 cm (girls), HDL < 50 mg/dL (boys) or < 40 mg/dL (girls). High ALT defined as > 24.1 U/L (boys) or > 17.7 U/L (girls).	Adolescents Boys: 2.6% Girls: 3.1%	Adolescents Boys: 0.54 Girls: 0.40
López‐Gil et al. [21] Chile	452 (185 boys, 267 girls)	10–17	Cross‐sectional (Growth and Obesity Chilean Cohort Study)	Handgrip strength was assessed with a Baseline 12‐0286 digital dynamometer, adjusted by sex and hand size. Two trials per hand were performed with 1‐min rest; the average of the highest values from both hands was used.	Continuous CMR score calculated as the sum of z‐scores for either: (i) WC to height ratio, insulin, triglycerides, HDL (×−1) and glycaemia; or (ii) WC, insulin, triglycerides, HDL (×−1) and glycaemia.	Children Boys: 31.0% Girls: 32.0%	Children Boys: 0.33 Girls: 0.40
Peterson et al. [14] USA	1326 (630 boys, 696 girls)	6–11	Cross‐sectional (NHANES 2011–2012)	Handgrip strength was measured with a Jamar hydraulic dynamometer (three trials of dominant hand; mean used).	Continuous CMR score calculated as the sum of z‐scores (standardized residuals regressed on age and race) for percentage of body fat, SBP, triglycerides, glucose and inverse HDL‐C.	Children Boys: 24.4% Girls: 26.7%	Children Boys: 0.45 Girls: 0.36
Ramírez‐Vélez et al. [23] Colombia	1950 (859 boys, 1091 girls)	10–17	Cross‐sectional (FUPRECOL)	Handgrip strength was measured using a Takei TKK 5401 dynamometer (two trials per hand; average used).	CMR index calculated as the sum of age‐ and sex‐standardized z‐scores for WC, TG, HDL‐C (×−1), glucose, SBP and DBP.	Children Boys: 16.0% Girls: 15.9% Adolescents Boys: 15.9% Girls: 16.0%	Children Boys: 0.38 Girls: 0.36 Adolescents Boys: 0.45 Girls: 0.44
Present study Spain	1124 (603 boys, 521 girls)	9–11	Cross‐sectional (MOVI‐2)	Handgrip strength was measured with a TKK 5401 dynamometer (two trials per hand; highest value per hand recorded). The average of the highest values from both hands was used.	A CMR index was computed by summing the age‐ and sex‐standardized z‐scores of WC, the triglyceride‐to‐HDL cholesterol ratio, mean arterial pressure and fasting insulin levels.	Children Boys: 27.8% Girls: 26.5%	Children Boys: 0.38 Girls: 0.35

Abbreviations: BP, blood pressure; CMR, cardiometabolic risk; HDL, high‐density lipoprotein; HELENA, Healthy Lifestyle in Europe by Nutrition in Adolescence; KNHANES, Korea National Health and Nutrition Examination Survey; NHANES, National Health and Nutrition Examination Survey; TG, triglycerides; WC, waist circumference.

These studies were conducted in the United States [13, 14], Spain [19], Colombia [23], Chile [21], South Korea [20, 22] and across several European nations through the HELENA study [18]. CMR was defined either through a continuous score, derived from standardized markers such as WC, blood pressure, glucose, lipid profile and insulin, or categorically, based on criteria for metabolic syndrome such as the International Diabetes Federation (IDF) [20] or national adaptations [22] (Table 2).

Handgrip strength was assessed using portable dynamometers, most commonly the TKK 5101 Grip‐D, Jamar [14] or Baseline device [21]. While procedures were generally similar, minor variations existed across studies. Some instructed participants to perform the test standing with the arm fully extended [22], while others used a 90° elbow flexion position [13]. Typically, two or three trials per hand were performed, and the best or average value was recorded (Table 2).

#### 3.2.3 Risk of Bias and Applicability

The QUADAS‐2 assessment indicated generally low risk of bias across the included studies. However, due to the absence of a universally accepted definition of CMR in paediatric populations, all studies were rated as having ‘some concerns’ for the item assessing the accuracy of the reference standard in correctly classifying the target condition. Additionally, two studies were identified as having a potential selection bias [19, 23], as they did not employ random sampling methods in participant recruitment (Table S2).

#### 3.2.4 Results of Individual Studies and Synthesis of Results

Among children aged 6–12 years, girls showed the highest pooled sensitivity (77%; *I* ^2^ = 53.95%) and a moderate AUC of 0.81, indicating good diagnostic performance. However, specificity was low (53%) and highly heterogeneous (*I* ^2^ = 98.60%). The PLR and NLR were 1.64 and 0.42, respectively. Boys in the same age group exhibited slightly lower sensitivity (76%; *I* ^2^ = 22.96%) but higher specificity (69%; *I* ^2^ = 95.66%), with an AUC of 0.76, PLR of 2.48 and NLR of 0.35.

In adolescents aged 13–18 years, girls had a sensitivity of 76% (*I* ^2^ = 64.70%) and the highest specificity across all groups (84%; *I* ^2^ = 97.70%), resulting in the highest AUC (0.80), PLR (4.88) and the lowest NLR (0.28), suggesting strong diagnostic performance. Adolescent boys also showed favourable results, with 77% sensitivity (*I* ^2^ = 0.0%) and 70% specificity (*I* ^2^ = 94.45%). Their AUC was 0.79, with a PLR of 2.57 and an NLR of 0.33.

#### 3.2.5 Additional Analyses

As shown in Table 3, the optimal thresholds for handgrip strength normalized to body mass differed slightly across subgroups. Among children, the thresholds were 0.30 for girls and 0.39 for boys. In adolescents, they were 0.36 for girls and 0.42 for boys. These thresholds yielded Youden Index values ranging from 0.30 to 0.60, with the highest diagnostic discrimination observed in adolescent girls (Youden Index = 0.60).

**TABLE 3:** Pooled diagnostic performance indicators by sex and age group.

	Optimal threshold	Youden Index	Sensitivity (%)	*I* ^2^ (sensitivity)	Specificity (%)	*I* ^2^ (specificity)	AUC (95% CI)	PLR	NLR
**Children (6–12 years)**									
Girls	**0.30**	0.30	77	53.95	53	98.60	0.81 (95% CI: 0.79–0.83)	1.64	0.42
Boys	**0.39**	0.46	76	22.96	69	95.66	0.76 (95% CI: 0.74–0.78)	2.48	0.35
**Adolescents (13–18 years)**									
Girls	**0.36**	0.60	76	64.70	84	97.70	0.80 (95% CI: 0.77–0.83)	4.88	0.28
Boys	**0.42**	0.54	77	0.0	70	94.45	0.79 (95% CI: 0.76–0.82)	2.57	0.33

Abbreviations: AUC = area under the curve; *I* ^2^ = heterogeneity index; NLR = negative likelihood ratio; PLR = positive likelihood ratio.

*Note:* Optimal diagnostic thresholds for each sex and age group are highlighted in bold to facilitate clinical interpretation.

## 4 Discussion

Our study provides new evidence by proposing sex‐ and age‐specific thresholds for identifying CMR in children and adolescents, both in a large sample of Spanish youth and at a bigger level through a meta‐analytic approach. Specifically, the meta‐analysis identified optimal thresholds of normalized handgrip strength during childhood (6–12 years): 0.30 for girls and 0.39 for boys. In adolescence (older than 12 years), the thresholds were 0.36 for girls and 0.42 for boys, providing clinically relevant reference values for evaluating CMR by sex and age. Despite variability in specificity due to differences in study design, handgrip strength test protocol and diagnostic criteria, these thresholds provide a useful starting point for CMR screening in boys and girls. Nonetheless, the clinical implications of potential false positives should be considered in practice and weighed against the benefits of early detection and intervention, particularly in girls, where thresholds tended to show higher sensitivity but lower specificity.

The benefits associated with resistance training in children and adolescents are well established [24, 25]. Higher levels of muscular fitness during childhood and adolescence have been linked to a reduced long‐term CMR [3, 4], highlighting the potential of muscular fitness as an early health indicator. The association between muscular fitness and reduced CMR in youth may be partly explained by increased insulin sensitivity [26], enhanced glucose metabolism [27] and lower systemic inflammation [28] linked to increased muscle mass and function. This evidence supports the use of handgrip strength as a valuable screening tool to identify children and adolescents at risk and to inform early preventive strategies [29]. However, the lack of widely accepted health‐related thresholds for muscular fitness has been identified as one of the major barriers to the implementation of national fitness surveillance systems [5, 30]. Our systematic review and meta‐analysis addressed this gap by synthesizing evidence from nine studies conducted across diverse settings, providing pooled estimates of diagnostic accuracy by sex and age group.

Pooled data from the meta‐analysis revealed that adolescent girls showed the strongest diagnostic performance, while children presented lower specificity and greater heterogeneity, particularly among girls. This pattern may be partly explained by developmental variability in body composition and hormonal maturation, which influence both cardiometabolic profiles and the performance on muscular fitness measures [31]. Younger children typically show greater interindividual variability and less stable associations between muscular fitness and metabolic markers, which may limit the diagnostic accuracy of cut‐offs in this age group [32]. Importantly, the meta‐analysis demonstrated that optimal normalized handgrip strength thresholds vary by sex and developmental stage, ranging from 0.30 to 0.42. These values reflect physiological differences and emphasize the need for age‐ and sex‐specific standards. Although a previous meta‐analysis by Lee et al. [6] provided valuable insights into handgrip strength thresholds for CMR, certain methodological limitations warrant discussion. Particularly, their analyses relied heavily on repeated use of the KNHANES dataset in three studies, all drawing from the same time period (2014–2018), potentially affecting the generalizability of the findings. Notably, overreliance on a single dataset may overestimate internal validity and precision while reducing external validity, thereby limiting the clinical utility of the proposed thresholds [33].

## 5 Strengths and Limitations

This study presents several strengths. First, it combines original cross‐sectional data from a large sample of Spanish schoolchildren with a systematic review and meta‐analysis, enhancing both internal robustness and external applicability. Second, unlike previous investigations, it establishes sex‐ and age‐specific diagnostic thresholds, offering more precise and developmentally appropriate reference values.

Nevertheless, some limitations should be acknowledged. Considerable heterogeneity was observed across studies, particularly in specificity, which may be attributed to differences in study design, handgrip strength testing protocols and the varying definitions of CMR used. While some studies relied on standardized z‐score composites, others used clinical diagnostic criteria such as those proposed by the IDF or adapted national guidelines. This lack of uniformity complicates direct comparisons and limits the potential to conduct robust subgroup analyses or meta‐regressions. The application of the handgrip strength test was not standardized across studies, with differences in position, measurement devices and number of trials, potentially affecting comparability. Moreover, the cross‐sectional nature of the primary dataset precludes causal inferences. In two studies [13, 14], diagnostic accuracy metrics were estimated from summary data rather than directly reported, which may introduce estimation bias. Additionally, it is important to acknowledge that the method used to normalize handgrip strength may not be the most appropriate. In the present analysis, normalization decisions were based on the methodology used in the included studies. However, recent literature suggests that expressing normalized handgrip strength relative to height squared (i.e., handgrip strength/height^2^, in kg/m^2^) may offer a more valid adjustment based on allometric scaling, as it better accounts for body size dimensions associated with muscle strength [34]. Finally, the lack of a universally accepted definition of CMR in pediatric populations remains a challenge, contributing to variability in outcome measures.

## 6 Conclusion

Our study provides novel age‐ and sex‐specific diagnostic thresholds for Spanish children aged 8–11, a population often underrepresented in previous research. Additionally, it offers a synthesis of diagnostic metrics across age groups and sexes in both children and adolescents. These findings support the integration of handgrip strength assessment into early preventive strategies and public health policies, and suggest that standardized protocols, aligned with updated consensus guidelines [35], can be feasibly implemented in clinical and epidemiological settings.

## Consent

Informed consent was obtained from all the participants and their parents or legal guardians.

## Conflicts of Interest

The authors declare no conflicts of interest.

## Supporting information


**Table S1:** Electronic search strategies.
**Figure S1:** PRISMA flow diagram.
**Table S2:** Risk of bias assessment based on the quality assessment of diagnostic accuracy studies (QUADAS‐2) for included studies.

## Data Availability

Data that support the findings of this review are available upon request from the corresponding author. The data were not publicly available because of privacy or ethical restrictions.
