# Supplementary material for: Handgrip Strength Thresholds to Detect Cardiometabolic Risk in Youth: Cross‐Sectional Study and Meta‐Analysis
Source: J Cachexia Sarcopenia Muscle. 2025 Oct 20;16(5):e70091. doi: 10.1002/jcsm.70091 (PMC12536247; doi:10.1002/jcsm.70091)
Supplement: Supplementary file 1 — Table S1: Electronic search strategies. Figure S1: PRISMA flow diagram. Table S2: Risk of bias assessment based on the quality assessment of diagnostic accuracy studies (QUADAS‐2) for included studies. [file JCSM-16-e70091-s001.docx]

Electronic Supplementary Material

Handgrip strength thresholds to detect cardiometabolic risk in youth: cross-sectional study and meta-analysis

**eTable 1. Electronic search strategies.**

| eTable 1. Electronic search strategies. | | |
| --- | --- | --- |
| Database | Search strategy | n |
| PubMed (MEDLINE) | ("handgrip strength"[MeSH Terms] OR "grip strength" OR "hand strength") AND ("metabolic syndrome"[MeSH] OR "cardiometabolic risk" OR "insulin resistance" OR "blood pressure" OR "dyslipidemia") AND ("child"[MeSH] OR "adolescent"[MeSH] OR "pediatric") AND ("cut-off" OR "cut-off point" OR "threshold" OR "diagnostic threshold" OR "reference value" OR "criterion value") | 228 |
| EMBASE | ('cardiorespiratory fitness'/exp OR 'aerobic fitness' OR 'vo2 max' OR 'maximal oxygen uptake' OR 'peak oxygen consumption')  AND  ('metabolic syndrome'/exp OR 'cardiometabolic risk' OR 'insulin resistance'/exp OR 'blood pressure'/exp OR 'dyslipidemia'/exp)  AND  ('child'/exp OR 'adolescent'/exp OR pediatric)  AND  ('cut off' OR 'cut-off point' OR 'threshold' OR 'diagnostic threshold' OR 'reference value' OR 'criterion value') | 421 |
| Web of Science | TS=("cardiorespiratory fitness" OR "aerobic fitness" OR "VO2 max" OR "maximal oxygen uptake" OR "peak oxygen consumption")  AND  TS=("metabolic syndrome" OR "cardiometabolic risk" OR "insulin resistance" OR "blood pressure" OR "dyslipidemia")  AND  TS=("child" OR "adolescent" OR "pediatric")  AND  TS=("cut-off" OR "cut-off point" OR "threshold" OR "diagnostic threshold" OR "reference value" OR "criterion value") | 630 |
| SPORTDiscus | AB("cardiorespiratory fitness" OR "aerobic fitness" OR "VO2 max" OR "maximal oxygen uptake" OR "peak oxygen consumption")  AND  AB("metabolic syndrome" OR "cardiometabolic risk" OR "insulin resistance" OR "blood pressure" OR "dyslipidemia")  AND  AB("child" OR "adolescent" OR "pediatric")  AND  AB("cut-off" OR "cut-off point" OR "threshold" OR "diagnostic threshold" OR "reference value" OR "criterion value") | 4 |

**eMethod 1. Reasons for exclusion.**

Bekolli B, Ramadani L, Ramabaja Q, Rashiti N, Bjelica D, Gontarev S. Using the Relative Handgrip Strength in Identification of the Under-Aged of Female Gender Candidates Exposed at Risk in Developing of Sarcopenic Obesity. Sport Mont. 2024;22(2):77–83.

Reason: No information on the outcome of interest.

Fredriksen PM, Mamen A, Hjelle OP, Lindberg M. Handgrip strength in 6–12-year-old children: the health oriented pedagogical project (HOPP). Scand J Public Health. 2018;46(21 Suppl):54–60.

Reason: Thresholds of interest not reported.

Gontarev S, Jakimovski M, Georgiev G. Using relative handgrip strength to identify children at risk of sarcopenic obesity. Nutr Hosp. 2020;34(3):490–6.

Reason: No information on the outcome of interest.

Jung HW, Lee J, Kim J. Handgrip strength is associated with metabolic syndrome and insulin resistance in children and adolescents: Analysis of Korea National Health and Nutrition Examination Survey 2014–2018. J Obes Metab Syndr. 2022;31(4):334.

Reason: Duplicated.

Li Z, Wu C, Song Y, Li Y, Zhao X, Shang Y, et al. Weight-Specific Grip Strength as a Novel Indicator Associated With Cardiometabolic Risk in Children: The EMSNGS Study. J Clin Endocrinol Metab. 2025;110(3):624–33.

Reason: Thresholds of interest not reported.

Steffl M, Chrudimsky J, Tufano JJ. Using relative handgrip strength to identify children at risk of sarcopenic obesity. PLoS One. 2017;12(5):e0177006.

Reason: No information on the outcome of interest.

**eFigure 1. PRISMA flow diagram.**

**eTable 2. Risk of bias assessment based on the quality assessment of diagnostic accuracy studies (QUADAS-2) for included studies.**

| Study | Patient Selection (Risk of Bias) | Index Test (Risk of Bias) | Reference Standard (Risk of Bias) | Flow and Timing (Risk of Bias) | Overall Judgment |
| --- | --- | --- | --- | --- | --- |
| Castro-Piñero et al. (2019, HELENA) | Low | Low | Some concern | Low | Low |
| DeHondt et al. (2023) | Low | Low | Some concern | Low | Low |
| Lee et al. (2022) | Low | Low | Some concern | Low | Low |
| López-Gil et al. (2021) | Low | Low | Some concern | Low | Low |
| Ko and Kim (2021) | Low | Low | Some concern | Low | Low |
| Castro-Piñero et al. (2019, UP&DOWN) | High | Low | Some concern | Low | Low |
| Peterson et al. (2016) | Low | Low | Some concern | Low | Low |
| Ramírez-Vélez et al. (2017) | High | Low | Some concern | Low | Low |
| Present study | Low | Low | Some concern | Low | Low |
